# Mutational pattern off homologous recombination repair (HRR)‐related genes in upper tract urothelial carcinoma

**DOI:** 10.1002/cam4.6175

**Published:** 2023-06-30

**Authors:** Kaiwei Yang, Wei Yu, Huanhuan Liu, Feng Lou, Shanbo Cao, Huina Wang, Zhisong He

**Affiliations:** ^1^ Department of urology Peking University First Hospital Beijing China; ^2^ AcornMed Biotechnology Co., Ltd. Beijing China

**Keywords:** homologous recombination repair (HRR), molecular mechanism, prognosis, tumor immune profile, upper tract urothelial carcinoma (UTUC)

## Abstract

**Background:**

Homologous recombination (HR) repair (HRR) has been indicated to be a biomarker for immunotherapy, chemotherapy, and poly‐ADP ribose polymerase inhibitors inhibitors (PARPis). Nonetheless, their molecular correlates in upper tract urothelial carcinoma (UTUC) have not been well studied. This study aimed to explore the molecular mechanism and tumor immune profile of HRR genes and the relevance of their prognostic value in patients with UTUC.

**Materials and Methods:**

One hundred and ninety‐seven tumors and matched blood samples from Chinese UTUC were subjected to next‐generation sequencing. A total of 186 patients from The Cancer Genome Atlas were included. Comprehensive analysis was performed.

**Results:**

In Chinese patients with UTUC, 5.01% harbored germline HRR gene mutations, and 1.01% had Lynch syndrome‐related genes. A total of 37.6% (74/197) of patients carried somatic or germline HRR gene mutations. There was marked discrepancy in the mutation landscapes, genetic interactions, and driver genes between the HRR‐mut cohorts and HRR‐wt cohorts. Aristolochic acid signatures and defective DNA mismatch repair signatures only existed in individuals in the HRR‐mut cohorts. Inversely, the unknown signature (signature A) and signature SBS55 only existed in patients in the HRR‐wt cohorts. HRR gene mutations regulated immune activities by NKT cells, plasmacytoid dendritic cells, hematopoietic stem cell, and M1 macrophages. In patients with local recurrence, patients with HRR gene mutations had poorer DFS rates than patients with wild‐type HRR genes.

**Conclusions:**

Our results imply that the detection of HRR gene mutations can predict recurrence in patients with UC. In addition, this study provides a path to explore the role of HRR‐directed therapies, including PARPis, chemotherapy, and immunotherapy.

## Graphical abstract

The homologous recombination repair (HRR) mutations were associated with poor DFS. The HRR mutations had unique molecular features. Patients with HRR mutations had an elevated immunocompetence.

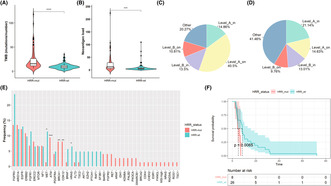

## 1 INTRODUCTION

In 2018, urothelial carcinoma (UC) was the 12th most common cancer, which originated in the upper tract (UT) and bladder.^1^ Upper urothelial carcinoma (UTUC) accounts for 5%–10% of all UC in Europeans.^2^ However, UTUC accounts for more than 30% in China.^3^ UTUC and UC of the bladder (UCB) had similar histologic appearance; however, UTUC shows a more advanced presentation than UCB, and more than 60% of patients have invasive disease at the time of diagnosis.^2^ Renal insufficiency is common in UTUC patients, and thoes patients are ineligible for certain treatments, although cisplatin‐based chemotherapy was currently the most effective chemotherapy agent for UC.^4^, ^5^ Immune checkpoint inhibitors (ICIs) are extensively applied to the management of UC; however, they have a limited response rate in UTUC patients.^6^ Therefore, improving the efficacy of treatment and exploring new therapeutic strategies are clinical challenges.

Homologous recombination repair (HRR) participates in DNA repair mechanisms, which might contribute to the reorganization of chromosomes, genome instability, and cell apoptosis.^7^ Recently, poly‐ADP ribose polymerase inhibitors (PARPis) have been approved for the treatment of metastatic prostate cancer carrying HRR gene mutations.^8^ Furthermore, HRR gene mutations are rendered sensitive to chemotherapy and immunotherapy.^9^, ^10^ Nonetheless, the molecular mechanisms and tumor immune profiles of HRR genes and their relevance to the response to clinical therapy in UTUC remain to be clarified. This knowledge is essential for designing biomarker for guiding combination therapies for UTUC patients.

The aim of this study was to comprehensively characterize the landscape of HRR gene mutations and the immune profile in UTUC patients. In addition, we explored the relationship between HRR status and clinical outcomes.

## 2 METHOD

### 2.1 Patient enrollment and study design

A total of 197 Chinese UTUC patients were registered at Peking University First Hospital between January 2019 and April 2021. All participants provided written the informed consent. The Ethical Committee of Peking University First Hospital (No.2021.189) approved this study. The Cancer Genome Atlas (TCGA) data from 186 patients were obtained from the cBioPortal data portal.

### 2.2 Next‐generation sequencing

QIAamp Genomic DNA kit (QIAGEN) was used to extract DNA from tissue and matched blood, based on our previously reported method.^1^ ctDNA was isolated from at least 2 mL plasma with a QIAamp Circulating Nucleic Acid kit (QIAGEN). Illumina standard library construction instructions (Illumina, Inc.) were performed to create libraries. Illumina HiSeq2500. Next‐generation sequencing (NGS) was applied to sequence using the Acornmed 808 panel with 808 cancer‐related genes. Burrows–Wheeler alignment (BWA) tool was performed to align the sequence reads to the reference genome (GRCh37).^11^ GATK software served as local realignment and base quality score recalibration. Somatic mutations were identified by MuTect2.^12^ Copy number variants (CNV) were identified by CONTRA software.^13^

### 2.3 Analyzing mutational signatures

Mutational signature analysis was resolved the SNVs into 96 base substitution types for each sample using Bayesian NMF.^1^ The discovered signatures were compared with COSMIC signatures according to the hierarchical clustering of cosine similarity among these signatures. If values of similarity for discovered signatures were <0.80, this signature was defined as the new signature.^14^

### 2.4 Inferring the clone or subclone mutation

ABSOLUTE was used to detect the clonal or subclone mutation using the value of the cancer cell fraction (CCF) representing the fraction of tumor cells in the sample which carry the mutation.^1^ We defined the *Pr*(*clonal*) representing the probability that a mutation is clonal. If the Pr(clone) was >0.5 and CCF was >0.9, the mutation was defined as a clone, otherwise as a subclone.^15^

### 2.5 Gene set enrichment analysis (GSEA)

The “edgeR” package was performed to identify the differentially expressed genes (DEGs) (fold change >1.5, FDR <0.05, and TPM >1). Gene set enrichment analysis (GSEA) was analyzed by the “clusterprofiler” and “ggplot2” packages using DEGs.

### 2.6 Immune cell abundance and immune signature genes

Immune cell abundances were evaluated by the TIMER 2.0 web (http://timer.cistrome.org/) in the TCGA cohort data. The immune signature genes in the tumor microenvironment (TME) were calculated from RNA sequencing (RNA‐seq) data used in previous studies.^16^

### 2.7 Neoantigen predictions

HLA typing was acquired from normal DNA using OptiType. Peptide–MHC affinity for half maximal inhibitory concentration (IC_50_) values were predicted using pVACtools. Mutated peptides with a binding affinity of IC_50_ < 500 nM were regarded as candidate neoantigens. For patient‐specific neoantigen prediction, all the peptides with 8–11 amino acids including point mutations, in‐frame and frameshift insertions and deletions, and gene fusion. Neoantigen load is defined as the total number of candidate neoantigens per patient.

### 2.8 Statistical analysis

R software was used to perform the statistical analysis. Two categorical variables were calculated using Fisher test. Two continuous variables were performed by the *t*‐test or Mann–Whitney test. False discovery rate (FDR) correction was carried out. Probability values were derived from two‐sided test, and differences for which *p* < 0.05 were considered significant.

## 3 RESULTS

### 3.1 Patient demographics

One hundred ninety‐seven Chinese UTUC patients were recruit in this study, including 176 (89.3%) tumor tissues and 21 (10.7%) ctDNA samples. In terms of tumor location, most patients harbored a tumor in the pelvis (53%) or ureter tract (41%) (Table S1). Clinical data were not associated with HRR status. Clinical characteristics are summarized in Table S1.

### 3.2 Characterization of pathogenic germline HRR gene mutations and Lynch syndrome‐related genes

**FIGURE 1:**
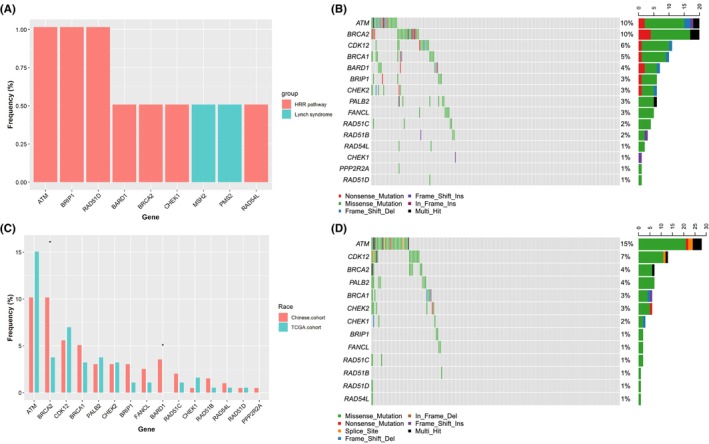
Landscapes of frequently mutated homologous recombination repair (HRR) genes in upper tract urothelial carcinoma (UTUC) patients. (A) Landscapes of pathogenic germline HRR gene mutations (red bars) and Lynch syndrome‐related genes (blue bars). (B) The most frequent somatic HRR genes in Chinese patients with UTUC. (C) Difference in the frequency of HRR gene mutations between individuals in the Chinese UTUC cohort (red bars) and The Cancer Genome Atlas (TCGA) UTUC cohort (blue bars). (D) The most frequent somatic HRR genes in TCGA patients with UTUC.

The basis of hereditary breast cancer and ovarian cancer is the haploinsufficiency of factors that controls the stability of the genome. One of the important ways is HRR genes.^17^ Germline mutations in mismatch repair were associated with Lynch syndrome (LS), which increased the risk of hypermutation and microsatellite instability.^18^ Thus, we explored the characterization of pathogenic germline HRR gene mutations and LS‐related genes in Chinese UTUC (our cohort). Seven pathogenic germline HRR gene mutations were identified in 5.08% (10/197) of Chinese patients, and the most frequently mutated HRR genes were *ATM* (1.01%), *BRIP1* (1.01%), and *RAD51D* (1.01%) (Figure 1A). Regarding the LS‐related genes, only *MSH2* (0.51%) and *PMS2* (0.51%) were discovered in 1.01% (2/197) of patients (Figure 1A). None of the patients had both HRR gene mutations and LS‐related gene mutations.

Then, we compared our cohort to published reports, including data from Fudan University Shanghai Cancer Center (FUSCC) and Memorial Sloan Kettering Cancer Center (MSKCC).^19^ There were a similar frequency of germline HRR gene mutations among our cohort (5.08%, 10/197), FUSCC cohort (6.47%, 20/309), and MSKCC cohort (6.14%, 7/114). Whereas, the MSKCC cohort (7.89%, 9/114) had a higher frequency of germline LS‐related genes than those in our cohort (1.01%, 2/197) and FUSCC (2.59%, 8/309) (*p* < 0.05). Compared to the MSKCC cohort, our cohort had a lower frequency of germline *MSH2* mutation (0.51% vs. 5.26%, *p* = 0.020), *MSH6* mutation (0 vs. 1.75%, *p* = 0.064), and *CHEK2* mutation (0 vs. 1.75%, *p* = 0.064) (Figure S1). Our cohort had higher frequencies of germline *ATM* mutations (1.01% vs. 0%, *p* = 0.076) and *BRCA2* mutations (0.51% vs. 2.59%, *p* = 0.084) than the FUSCC cohort (Figure S1).

### 3.3 Landscapes of somatic HRR gene mutations in UTUC patients

A total of 130 nonsilent somatic HRR gene mutations were noted in 34.5% (68/197) of Chinese patients, including benign, variant of uncertain significance and includes benign or variant of uncertain significance. Additionally, among all patients with HRR gene mutations, the percentage of the pathogenic HRR gene mutations reached 60.3% (41/68). Four patients carried both somatic and germline HRR gene mutations. Thus, 37.6% (74/197) of patients carried somatic or germline HRR gene mutations. The most frequent somatic HRR gene mutations were *ATM* (10.2%), *BRCA2* (10.2%), and *CDK12* (5.6%) (Figure 1B). There were similar nonsilent somatic HRR gene mutations in the TCGA cohorts. A total of 145 somatic HRR gene mutations were mapped among 28.5% (53/186) of the patients. The most frequent HRR mutations were *ATM* (15.1%), *CDK12* (7.0%), and *BRCA2* (3.8%) (Figure 1D). Interestingly, compared to TCGA cohorts, Chinese cohorts (our cohorts) had more frequent mutations for *BRCA2* (10.2% vs. 3.8%) and *BARD1* (3.6% vs. 0) (Figure 1C).

### 3.4 Differences in gene profiles and pathways among HRR mutation statuses

**FIGURE 2:**
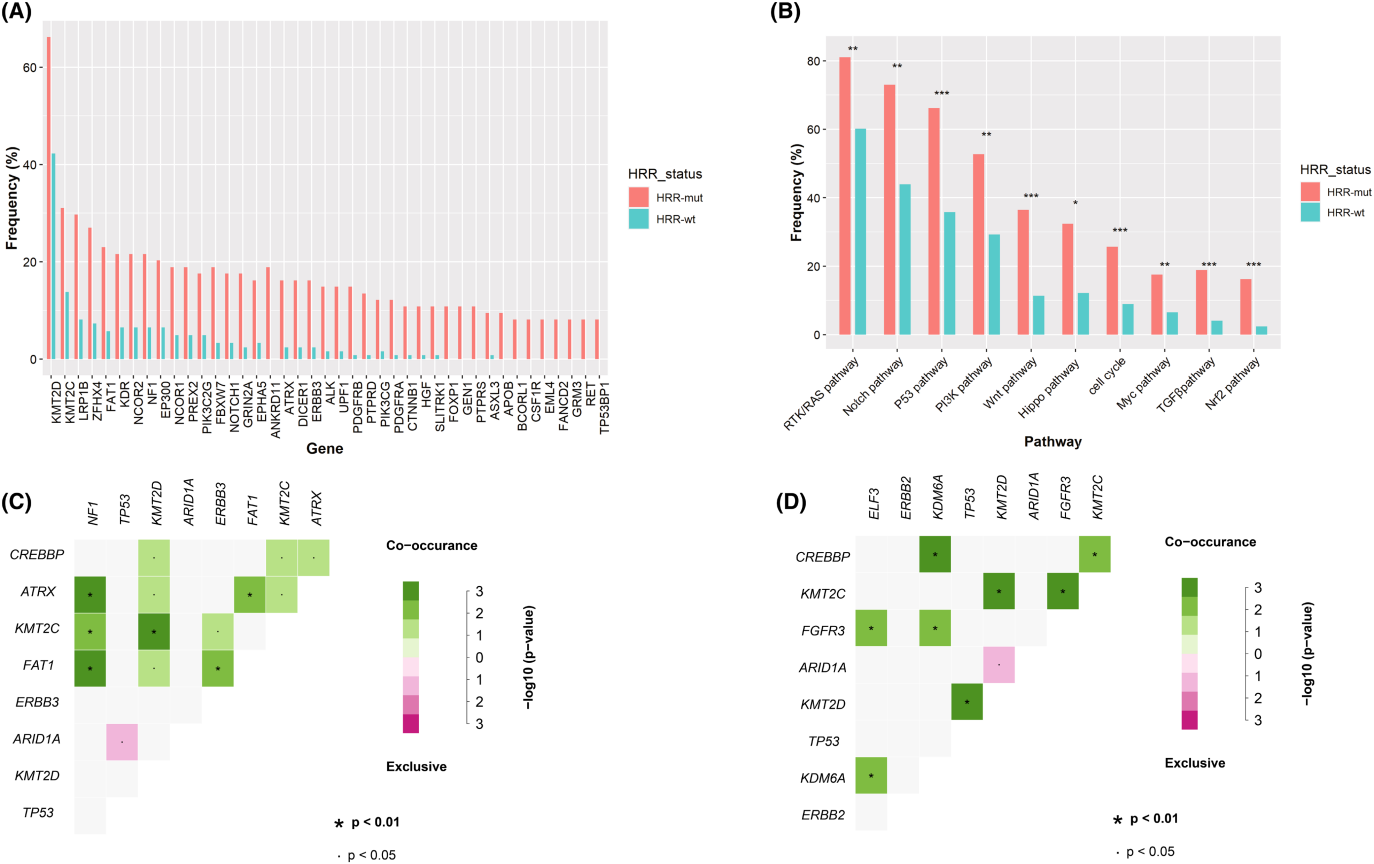
Differences in molecular mechanisms between the homologous recombination repair (HRR)‐mut cohorts and HRR‐wt cohorts. Differences in (A) gene profiles and (B) oncopathways between the two cohorts. Correlation between observed and expected co‐occurrence of mutations for the (C) HRR‐mut cohort and (D) HRR‐wt cohort.

To understand the patterns of somatic mutation in both the HRR‐mut and HRR‐wt cohorts, somatic mutations were identified. A total of 3315 nonsynonymous mutations were discovered in 74 patients in the HRR‐mut cohort, ranging from 4 to 463 mutations. A total of 1430 nonsynonymous mutations existed in 123 patients in the HRR‐wt cohort, ranging from 0 to 115 mutations (Table S2). Fifty‐one genes were markedly discrepancy between the two cohorts, and in HRR‐mut cohorts was higher the mutation frequency compared to that in the HRR‐wt cohorts (FDR < 0.05, Figure 2A, Figure S2). In addition, the most common signaling pathways enriched in both cohorts were the RTK/RAS pathway, Notch pathway, and p53 pathway (Figure 2B). Furthermore, HRR‐mut groups were more prone to harbor mutations in genes related to oncogenic pathways than HRR‐wt groups (Figure 2B).

### 3.5 Alterations in genetic interactions in various HRR status

To further explore whether HRR genes alter genomic patterns, we analyzed the co‐occurrence and mutual exclusion. In the HRR‐mut and HRR‐wt groups, the *ARID1A* and *TP53* mutations were mutually exclusive (Figure 2C,D). In addition, *CREBBP* mutations were co‐occurred with *KDM6A* and *KMT2C* mutations, and *KMT2C* were associated with *KMT2D* mutations in both the HRR‐mut and HRR‐wt cohorts (Figure 2C,D). Surprisingly, *ATRX* mutations only were significantly associated with *CREBBP*, *NF1*, *KMT2D*, *FAT1*, and *KMT2C* mutations in the HRR‐mut cohort (Figure 2C). In contrast, *FGFR3* mutations only related with *ELF3*, *KDM6A*, and *KMT2C* mutations in the HRR‐wt cohort (Figure 2D).

### 3.6 Relationship between mutational signature and HRR status

**FIGURE 3:**
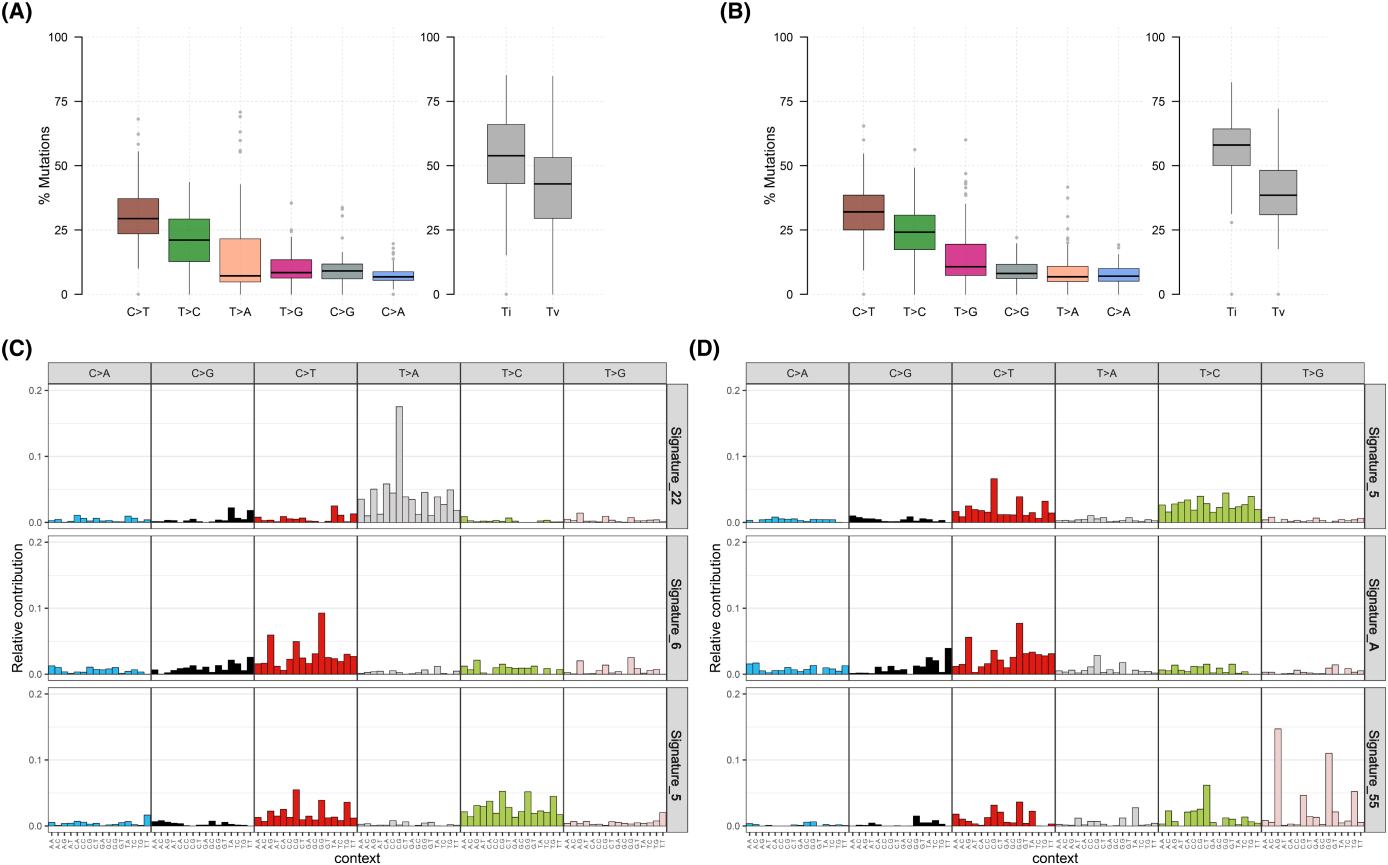
Dominant mutational signatures between upper tract urothelial carcinoma (UTUC) and UC of the bladder (UCB). The bar plot shows each type of transition or transversion in (A) homologous recombination repair (HRR)‐mut cohorts and (B) HRR‐wt cohorts. Mutational signatures in (C) HRR‐mut cohorts and (D) HRR‐wt cohorts.

To further explore the molecular characteristics of different HRR statuses in UTUC, we performed mutational signature and somatic substitution analyses. In patients with UTUC, transitions were major DNA substitution mutations, which are independent of HRR status (Figure 3A,B). In addition, C to T (C>T) substitutions were the predominant type in both the HRR‐mut cohort and HRR‐wt cohort (30% and 35%, respectively). Regarding the mutational signature, only signature SBS5 for the clock‐like signature existed in both cohorts. Furthermore, signature SBS22 (exposure to aristolochic acid [AA]) and signature SBS6 (defective DNA mismatch repair) were only observed in the HRR‐mut cohort (Figure 3C). Conversely, novel signature A and signature SBS55 only existed in individuals in the HRR‐wt cohort (Figure 3D).

### 3.7 Inference of clone mutation and driver mutation

**FIGURE 4:**
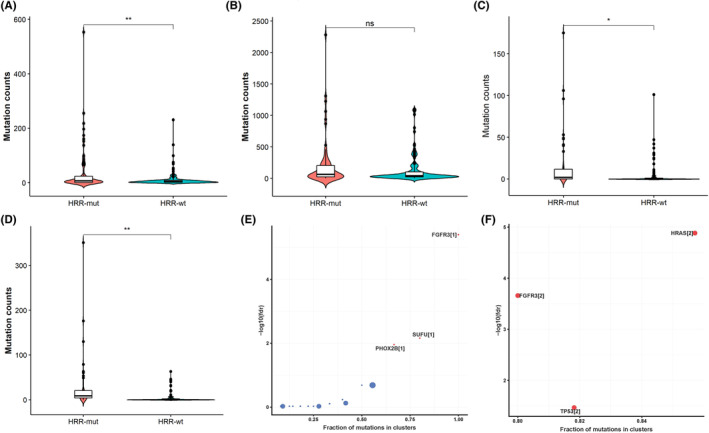
Differences in clonal mutation counts and driver genes between the HRR‐mut and HRR‐wt cohorts. (A) Comparison of clonal mutation counts for all somatic mutations between patients in the homologous recombination repair (HRR)‐mut and HRR‐wt cohorts. (B) Comparison of subclonal mutation counts for all somatic mutations between the HRR‐mut and HRR‐wt cohorts. (C) Comparison of nonsilent clonal counts between the HRR‐mut and HRR‐wt cohorts. (D) Comparison of nonsilent subclonal counts between the HRR‐mut and HRR‐wt cohorts. (E) Identification of driver genes in the HRR‐mut cohort. (F) Identification of driver genes in the HRR‐wt cohort.

To explore the differences in clonal evolution between the two cohorts, we compared and analyzed the clones and subclones of the two cohorts. Integrated with all somatic mutations, 2858 clonal mutations and 14,882 subclonal mutations were noted in 83.8% (62/74) of patients with HRR gene mutations. In addition, 1440 clonal mutations and 16,145 subclonal mutations were noted in 96.7% (119/123) of patients without HRR gene mutations HRR‐mut cohort had higher clonal counts (*p* = 0.008) than HRR‐wt cohort (Figure 4A). There were similar subclonal counts between the HRR‐mut and HRR‐wt cohorts (Figure 4B). Subsequently, we evaluated nonsilent clonal mutations, which may take part in carcinogenesis. A total of 892 nonsilent clonal mutations and 1663 nonsilent subclonal mutations were discovered in 43.9% 83.8% (62/74) of patients with HRR gene mutations. A total of 447 nonsilent clonal mutations and 540 nonsilent subclonal mutations were noted in 43.9% (54/123) of patients without HRR gene mutations. Furthermore, we discovered that HRR‐mut cohort harbored an elevated clonal counts (*p* = 0.022, Figure 4C) and subclonal counts (*p* = 0.002) compared to HRR‐wt cohort (Figure 4D). Whereafter, to explore whether different HRR statuses presented different carcinogenic mechanisms, we analyzed clonal driver genes using clonal nonsilent mutation. We discovered that various HRR status had different driver genes. *FGFR3*, *SUFU*, and *PHDX2B* mutations were the driver genes for the HRR‐mut cohort (Figure 4E). Inconsistently, the driver genes were *FGFR3*, *TP53*, and *HRAS* in the HRR‐wt cohort (Figure 4F).

### 3.8 Identification of DEGs and GSEA associated with HRR status

To explore the relationship between HRR status and transcriptomic signatures, we compared the difference in the whole transcriptome between the HRR‐mut and HRR‐wt cohorts in the TCGA cohort. 410 DEGs were identified, including 221 upregulated DEGs and 189 downregulated DEGs (Figure S3A). In addtion, GSEA of the HRR‐mut cohort against the HRR‐wt cohort revealed HRR gene mutation‐related biological signaling pathways. Ribosome (Figure S3B) and human papillomavirus infection signaling pathways (Figure S3C) were significantly enriched in the HRR‐mut cohort.

### 3.9 HRR gene mutations regulated immune activity

**FIGURE 5:**
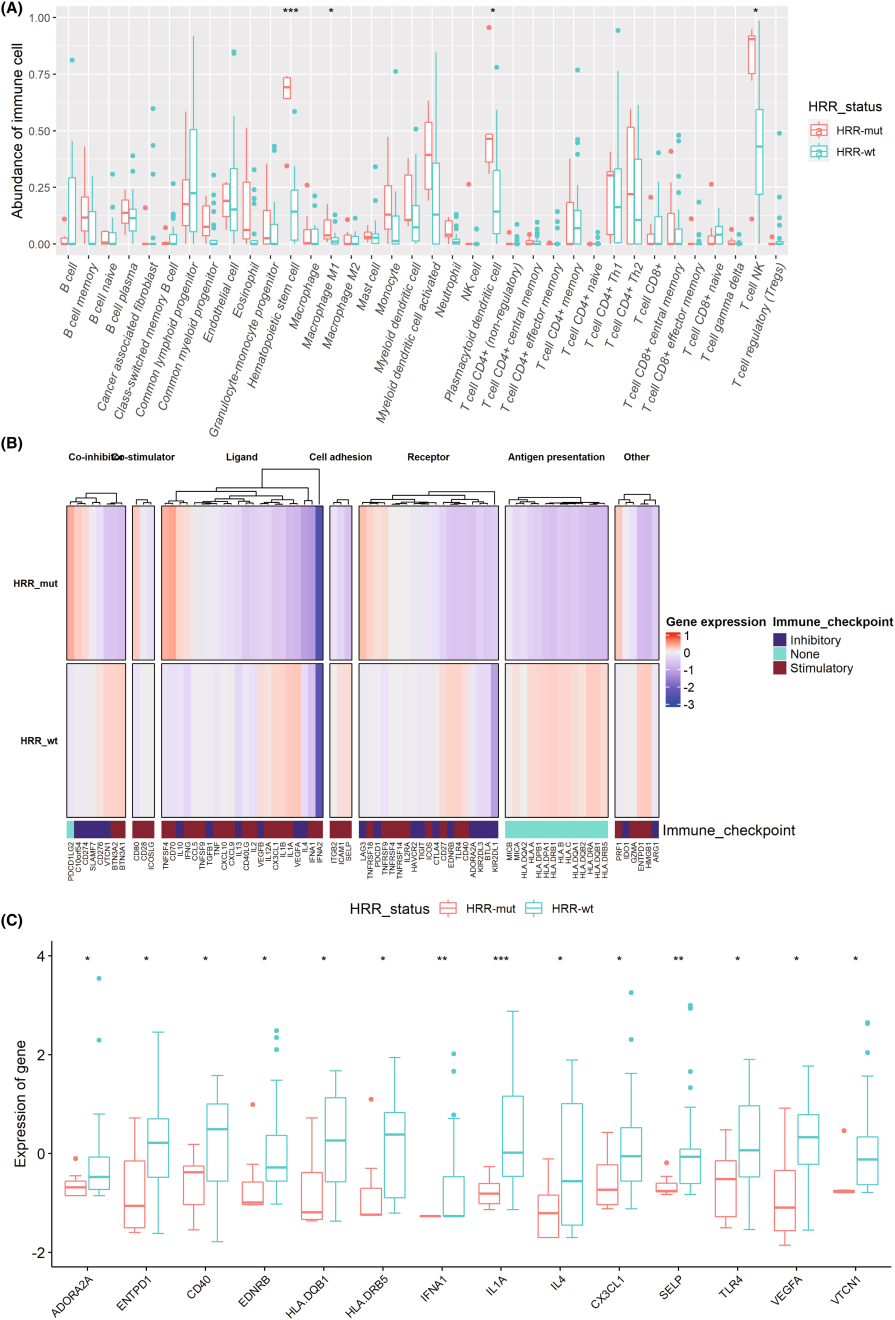
Homologous recombination repair (HRR) status was associated with immune infiltration. (A) The relationship between immune cell abundance and HRR status. (B) HRR status exhibits distinct expression patterns of immunoregulatory genes. The average *z* score of each immune‐regulatory gene was obtained. (C) Significantly differentially expressed genes between the HRR‐mut cohort and HRR‐wt cohort.

To further explore the role of HRR mutations in immune activity, we analyzed the relationship between HRR status and immune cells. Interestingly, patients with HRR gene mutations had elevated NKT cells (*p* = 0.043), plasmacytoid dendritic cells (*p* = 0.013), Hematopoietic stem cell (*p* < 0.001), and M1 macrophages (*p* = 0.018) (Figure 5A).

### 3.10 Immune regulatory gene expression profiles associated with HRR mutation status

To further explore how HRR gene mutations affect immune cell infiltration, we analyzed the relationship between HRR status and 77 immune regulatory genes. Interestingly, HRR‐mut cohort had lower expression of *ADORA2A* (*p* = 0.036), *ENTPD1* (*p* = 0.049), *CD40* (*p* = 0.049), *EDNRB* (*p* = 0.049), *HLA‐DQB1* (*p* = 0.022), *HLA‐DRB5* (*p* = 0.049), *IFNA1* (*p* = 0.004), *IL1A* (*p* < 0.001), *CX3CL1* (*p* = 0.030), *SELP* (*p* = 0.003), *TLR4* (*p* = 0.040), *VEGFA* (*p* = 0.033), and *VTCN1* (*p* = 0.011) than those in HRR‐wt cohort (Figure 5B,C).

### 3.11 HRR mutations predict immunotherapy feasibility

To explore the predictive value of HRR gene mutations for immunotherapy, we assessed the correlation between HRR gene mutations and immunotherapy markers. The TMB was defined as the number of somatic, coding, and indel mutations and base substitutions per megabase (Mb) of the genome examined, including splicing variants, synonymous mutations, nonsynonymous mutations, in‐frame mutations and frameshift mutations. neoantigen load.

**FIGURE 6:**
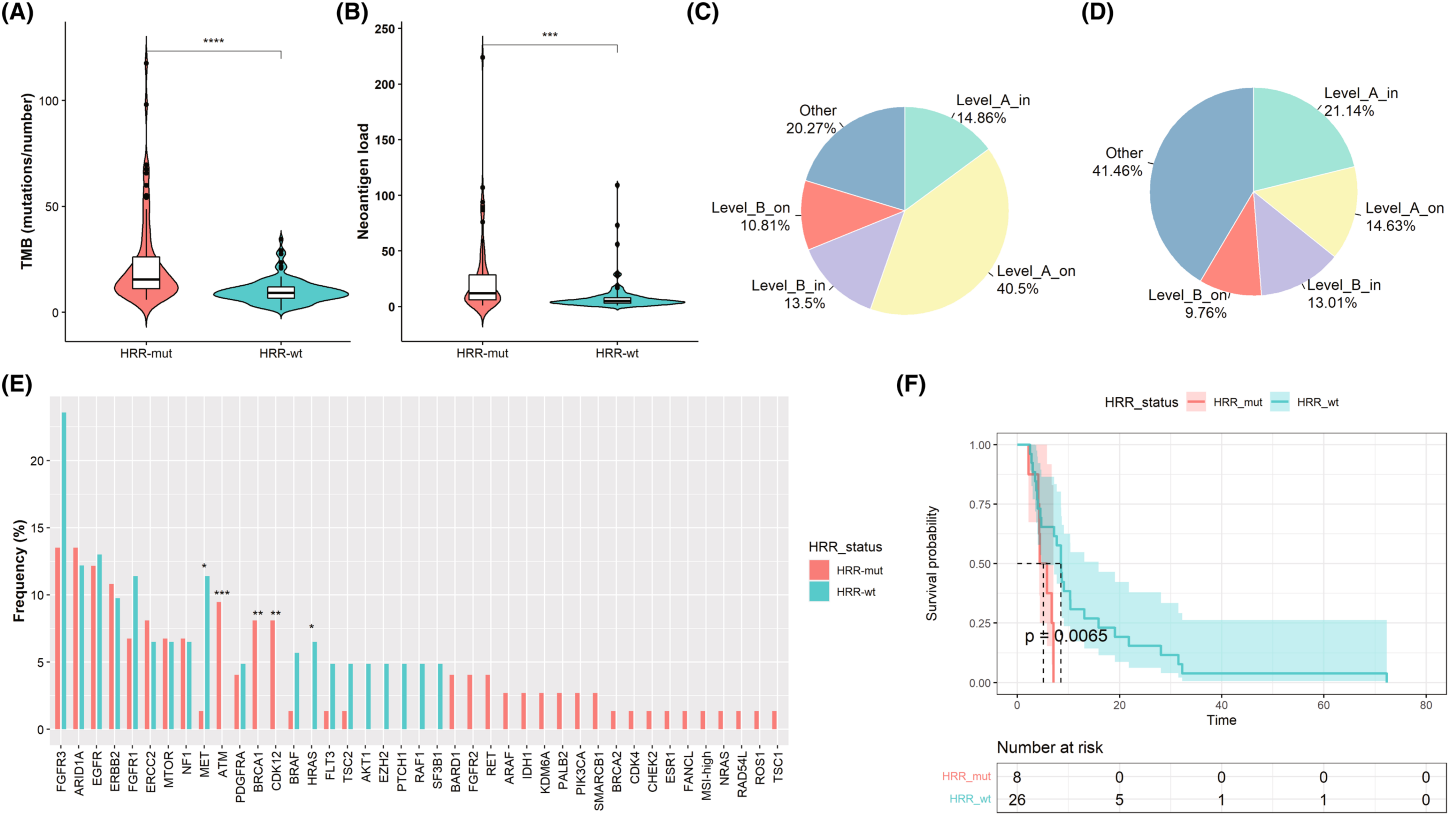
Relationship between homologous recombination repair (HRR) gene mutations and UTUC treatment. (A) Comparison of the TMB in patients in the HRR‐mut and HRR‐wt cohorts. (B) Comparison of the neoantigen burden in the HRR‐mut and HRR‐wt cohorts. (C) Distribution of the levels of actionable alterations in the HRR‐mut cohort. (D) Distribution of the levels of actionable alterations in UCB. (E) Distribution of targeted genes and drugs in the HRR‐mut and HRR‐wt cohorts. Level A, which corresponded to Levels 1 and 2 in OncoKB, represents the presence of biomarkers with either an approved therapy or guidelines, and level B, which corresponded to Levels 3 and 4 in OncoKB, represents biomarkers with strong biological evidence or clinical trials indicating that they are actionable. In‐label indicates a treatment registered by federal authorities for bladder cancer, whereas on‐label indicates a registration for other tumor types. (F) Disease‐free survival (DFS) stratified by HRR mutation status cohort.

Interestingly, HRR mutations were related to high TMB (*p* < 0.001, Figure 6A) and neoantigen load (*p* = 0.001, Figure 6B). HRR gene mutations were not correlated with CNV counts (*p* = 0.439, Figure S4A) or PD‐L1 expression (*p* = 0.181, Figure S4B).

### 3.12 Assessment of clinical actionability

To determine whether there are distinctions in targeted therapies for different HRR states and whether combination therapy can be used in patients with HRR mutations, we assessed the clinical actionability by OncoKB. 66.5% (131/197) patients had at least one clinical actionability, including 79.7% (59/74) of patients with HRR gene mutations and 58.5% (72/123) of patients with wild‐type HRR genes. HRR‐mut cohort harbored a higher frequency of clinical actionability than HRR‐wt cohort (*p* < 0.001), which may suggest that patients with HRR gene mutations were inclined to benefit from targeted therapy. In addition, HRR‐mut cohort had more mutations at Level A than those in HRR‐wt cohort (55.4% vs. 35.8%, *p* < 0.001, Figure 6C,D). Compared to the HRR‐wt cohort, the HRR‐mut cohort had higher *ATM* (*p* = 0.001), *BRCA1* (*p* = 0.002), and *CDK12* (*p* = 0.002) and lower *HRAS* (*p* = 0.026) and *MET* (*p* = 0.011) (Figure 6E).

### 3.13 HRR‐related disease‐free survival (DFS) in UTUC patients

To evaluate the prognostic effect of HRR mutations in UTUC, we explored the relationship between HRR mutations and clinical solution using TCGA data. In patients with local recurrence, patients with HRR gene mutations had poorer DFS than patients without HRR mutations (*p* = 0.007, Figure 6F). The median OS was not reached. Surprisingly, HRR status was not related to DFS (Figure S4C) or OS (Figure S4D) in patients receiving adjuvant chemotherapy.

## 4 DISCUSSION

UTUC is an aggressive disease with a high rate of recurrence and progression, but the clinical decision for UTUC has been extrapolated on account of UCB. In addition, due to the relative rarity for UTUC, the availability of immunotherapy and PARPis in patients with UTUC is unclear. To solve this problem, we analyzed the landscapes of HRR gene mutations in UTUC and demonstrated that 37.6% of patients may be eligible for PARPis and immunotherapy. Next, we determined that HRR mutations and wild‐type patients have different molecular and immune mechanisms. Furthermore, we believe that HRR mutations can guide relapse in patients with UTUC.

Characterization of germline HRR mutations is essential because of its implications for personalized treatment selection, familial testing, and risk prediction.^20^ We discovered that 5.08% of patients carried pathogenic germline HRR gene mutations, which may increase the risk of developing other cancers, such as *BRCA1/2* for breast cancer.^21^ The frequency of germline HRR gene mutations was generally similar in different regions, but Chinese UTUC patients harbored a higher frequency of somatic HRR gene mutations than Western UTUC patients, which may indicate that Chinese UTUC patients are more likely to benefit from immunotherapy, platinum‐based chemotherapy, and PARPis. In addition, there is a spectrum of germline or somatic HRR gene mutations between Chinese and Western UTUC patients. One explanation for these findings is the differences in the mechanism of HRR mutations in individuals in different racial cohorts.

Here, we divided Chinese UC patients into HRR‐mut and HRR‐wt cohort according to HRR mutation status and explored the molecular mechanism of HRR mutation. We found that patients with HRR mutations had more mutated genes and higher frequencies of oncogenic pathway mutations, which further confirms HRR mutation leading to increase mutation rate and genomic rearrangements.^22^ Drugs that target oncogenic pathways are currently under development or in clinical trials.^10^, ^23^ These results may indicate that the HRR mutations can be used to stratifying factor. In addition, differences in driving genes and genetic interactions between HRR‐mut cohorts and HRR‐wt cohorts suggested the different carcinogenic mechanism. In this study, HRR‐mut cohort had higher nonsilent with clonal and subclonal mutation numbers compared to HRR‐wt cohort, which implies more tumor heterogeneous in patients with HRR gene mutations. A high clonal number was related to bad prognosis in patients with glioblastoma and lung cancer.^24^, ^25^ Thus, we assumed that HRR mutations predicted a worse prognosis, which was needed further studies with larger patient cohorts and complete clinical data. Furthermore, Luo et al. reported that clonal HRR genes mutations were associated with prolonged survival in ovarian cancer with platinum‐based chemotherapy and all subclonal HRR mutated cases were resistant to platinum.^26^ We hypothesized that only patients with clonal HRR gene mutations may be predicted the efficacy of platinum‐based chemotherapy, immunotherapy, and PARPis in UTUC. Clinical trials that fully characterize the clonal status of HRR gene mutations will be required to confirm this hypothesis.

Mutational signatures can represent the multiformity and complicacy of somatic mutations.^1^ In this study, the AA signature and defective DNA mismatch repair signature were only observed in the HRR‐mut cohorts. These results may suggest that the AA signature and defective DNA mismatch repair signature can serve as novel readouts of HRD and contribute to defining UTUC patients with HRR mutations. AA exposure results in the appearance of an AA mutational signature.^27^ Thus, we hypothesized that administration of the herbal compound AA may mutate members of the HRR pathway and that patients taking AA may benefit from PARPis, immunotherapy, and platinum‐based chemotherapy. Furthermore, signature A and signature SBS55 only existed in the HRR‐wt cohorts, which may be used as a screening tool to screen patients with wild‐type HRR genes and avoid unnecessary treatment.

Immune cells participate in tumor progression and the immunotherapeutic response.^28^ In this study, HRR gene mutations enhanced the abundance of NKT cells, plasmacytoid dendritic cells, hematopoietic stem cell, and M1 macrophages. NKT cells play a dual role in the antitumor response. Type I NKT cells enhanced antitumor immunity based on direct tumor lysis, regulation of effector cells or immunosuppressive cells.^29^ Type II NKT cells induced tumor suppression by inhibiting tumor‐specific CD8^+^ T cells or Type I NKT cells.^30^ It has been reported that increased *IL‐4* and IL‐13 levels and AHR levels are dependent on type I NKT cells.^31^ Due to the lower *IL‐4* levels and higher abundance of NKT cells, we speculated that patients with HRR mutations had more Type II NKT cells than Type I NKT cells. Myeloid dendritic cell‐activated M1 macrophages play an antitumor role.^32^, ^33^ Inversely, plasmacytoid dendritic cells contribute to an immunosuppressive TME.^34^ Thus, we hold the opinion that HRR mutations increase both antitumor and immunosuppressive TMEs. In combination with survival outcomes, we speculated that HRR mutations are more likely to promote tumor growth in UTUC patients. We will further test our hypothesis in the future.

The expression of immune genes may further explain the regulation of HRR mutation on immune cells. We found that patients with HRR gene mutations had low expression of antigen presentation‐related genes such as CD40, HLA‐DQB1, HLA‐DRB5, IFNA1, and IL1A, which may indicate that HRR mutations reduce the antigen presentation response and increase plasmacytoid dendritic cell infiltration, thereby leading to tumor growth.^35^, ^36^, ^37^ In addition, we found HRR gene mutations were related to downregulated immunosuppressive genes, such as ADORA2A, ENTPD1 (CD39), EDNRB, VEGFA, and VTCN1 (VTCN1), which may mean that HRR gene mutations increase the infiltration of M1 macrophages by downregulating immunosuppressive genes, thus increasing the antitumor immune effect.^38^, ^39^, ^40^ Furthermore, we found low expression of dual‐acting genes (including CX3CL1 and TLR4) in the HRR‐mut group, whose role needs to be further explored in combination with its prognostic role.^41^, ^42^

Previous reports have demonstrated that the HRR pathway can predict immunotherapeutic or chemotherapeutic efficacy in some cancer types.^26^, ^43^ However, the predictive value of HRR mutations for UTUC remains unclear. Higher TMB levels and neoantigen loads have been proven to be targets for immunotherapy.^44^ In this study, patients in the HRR‐mut cohort had a higher TMB and neoantigen burden than other patients, that was consistent with other studies, which may suggest that HRR gene mutations are a predictive biomarker for immunotherapy and that immunotherapy combined with PARPis is feasible in UTUC patients.^44^, ^45^ Moreover, we found that HRR mutations can predict poor DFS in patients with local recurrence. We speculated that HRR gene mutations are prognostic biomarkers for recurrence in UTUC.

The study has several limitations: First, the results are limited due to the lack of transcriptome data and survival data from the Chinese cohort, which made it impossible to verify the findings obtained with the TCGA cohort in the Chinese cohort. Second, we did not include a medication regimen, so we could not accurately assess the predictive effect of HRR mutation on drug efficacy. Third, too few samples with RNA data may lead to some deviations in the analysis results. Last, due to the lack of TCGA raw data, we were unable to conduct clonal evolutionary analysis, etc., and verify our hypothesis. In the future, we will continue to collect samples and information to continuously verify the results.

### 4.1 Conclusion

HRR gene mutations exhibited a distinct molecular mechanism and immune profile. Our findings lay the foundation for identifying Chinese patients with UTUC who is suitable for ICIs, chemotherapy, and PARPis. In addition, our findings may provide insights into the biomarker development for further stratification of UTUC patients and a better, suitable approach to combination therapies in UTUC in clinical trials. In addition, our results may contribute to biomarker development for further stratification of UTUC patients and provide to better, suitable combination therapies for UTUC patients in clinical trials.

## AUTHOR CONTRIBUTIONS

**Kai‐Wei Yang:** Writing – original draft (lead); writing – review and editing (lead). **Wei Yu:** Formal analysis (equal); resources (equal). **Huanhuan Liu:** Methodology (equal); software (equal). **Feng Lou:** Conceptualization (equal); software (equal). **Shanbo Cao:** Conceptualization (equal); formal analysis (equal); validation (equal). **Huina Wang:** Conceptualization (equal); writing – original draft (equal); writing – review and editing (equal). **zhisong He:** Writing – original draft (equal); writing – review and editing (lead).

## FUNDING INFORMATION

This research did not receive any funding.

## CONFLICT OF INTEREST STATEMENT

The authors declared no competing interests.

## ETHICS STATEMENT

The Ethical Committee of Peking University First Hospital (No. 2021.189) approved this study. All participants provided written the informed consent and understood the content of the experiment and agree to publish the article.

## Supporting information


Figure S1.

Figure S2.

Figure S3.

Figure S4.

Table S1.

Table S2.

## Data Availability

The data may be available on request from the corresponding author.
