# Supplementary figures and images for: Mutational pattern off homologous recombination repair (HRR)‐related genes in upper tract urothelial carcinoma
Source: Cancer Med. 2023 Jun 30;12(14):15304–16. doi: 10.1002/cam4.6175 (PMC10417099; doi:10.1002/cam4.6175)

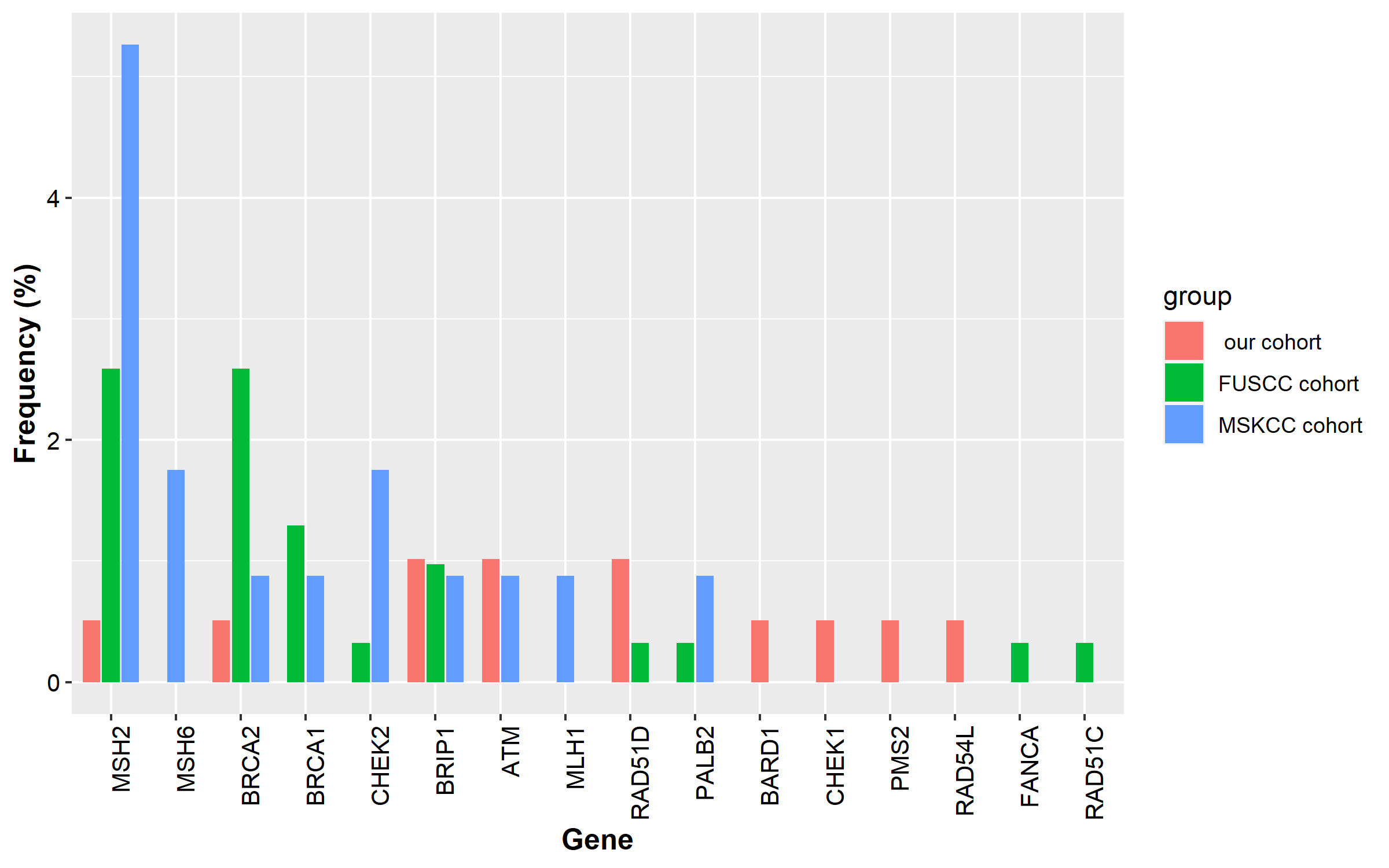

Supplement: Supplementary file 1 — Figure S1. [file CAM4-12-15304-s001.png]

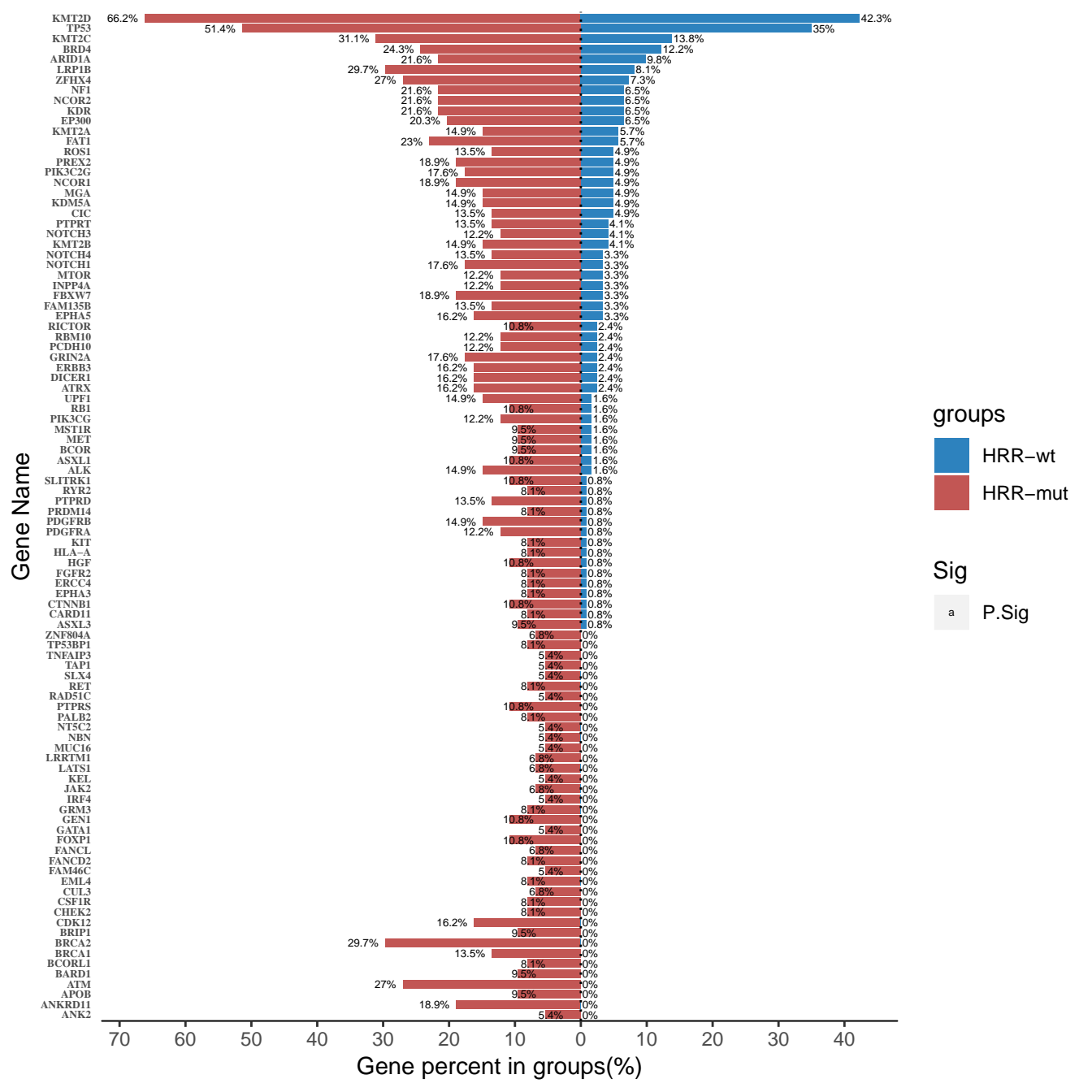

Supplement: Supplementary file 2 — Figure S2. [file CAM4-12-15304-s005.pdf]

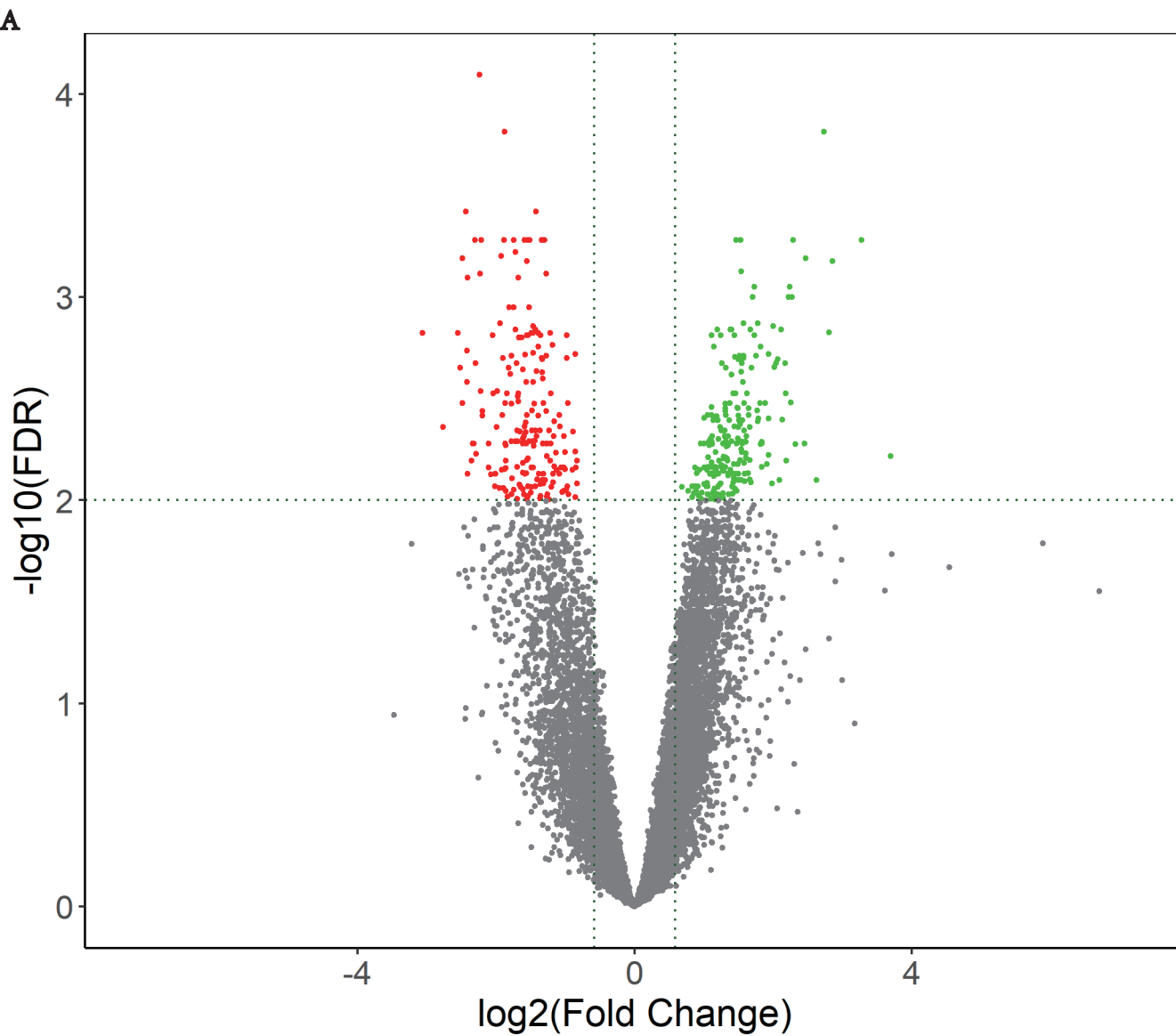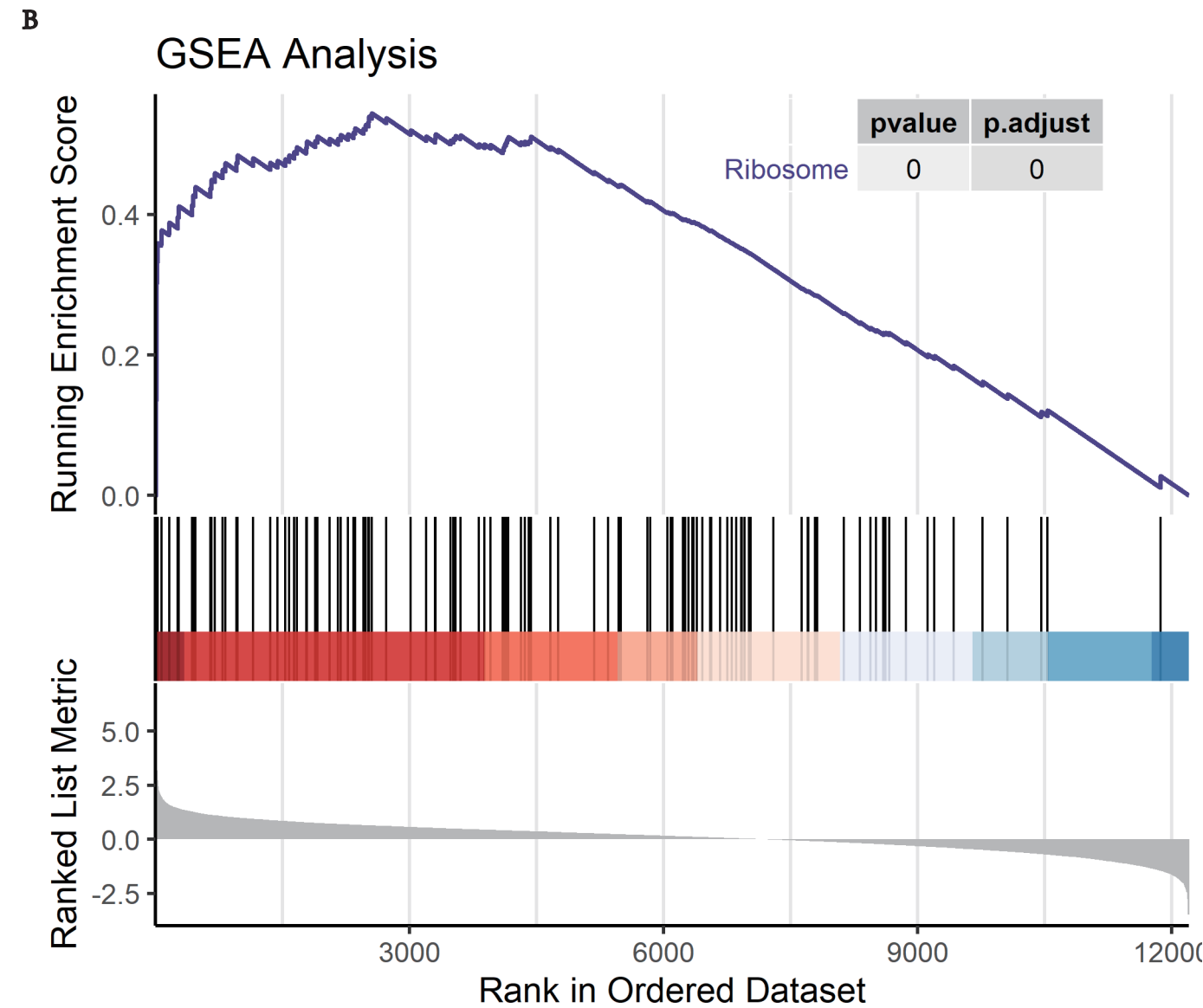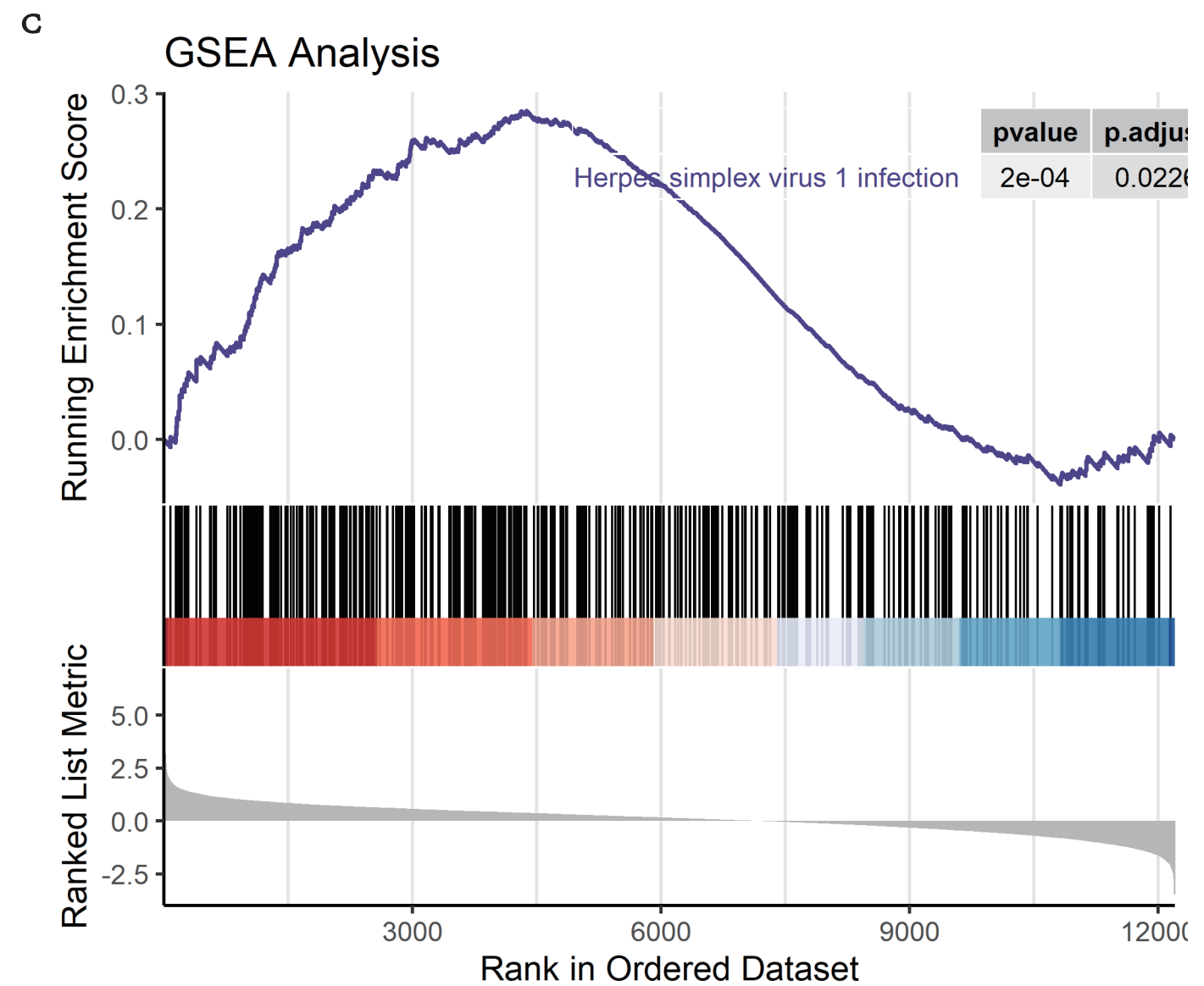

Supplement: Supplementary file 3 — Figure S3. [file CAM4-12-15304-s002.pdf]

**A**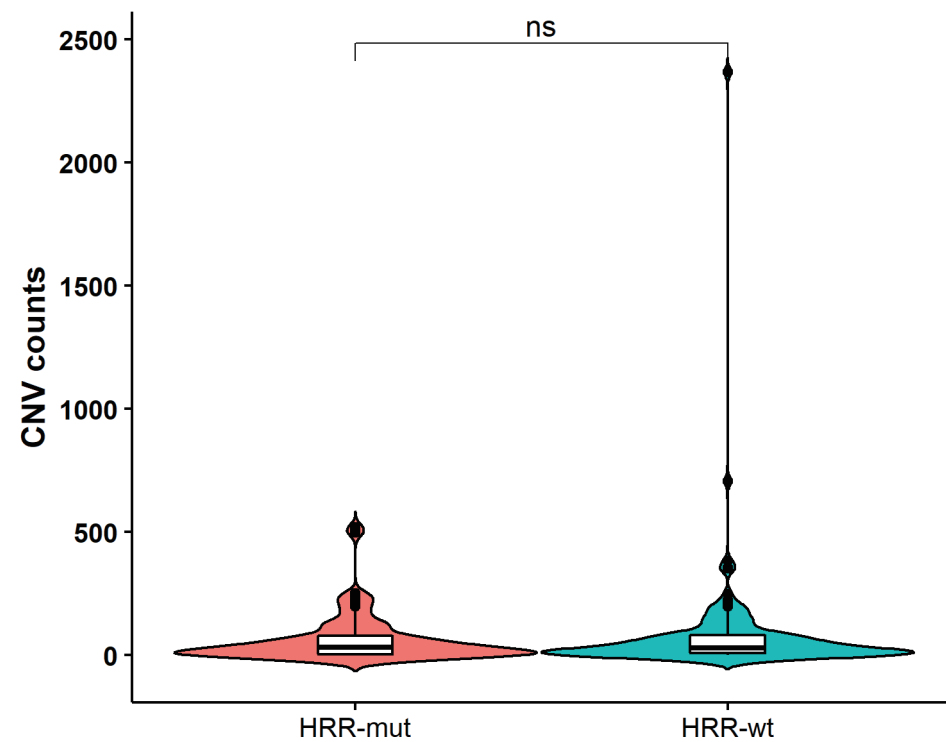**B**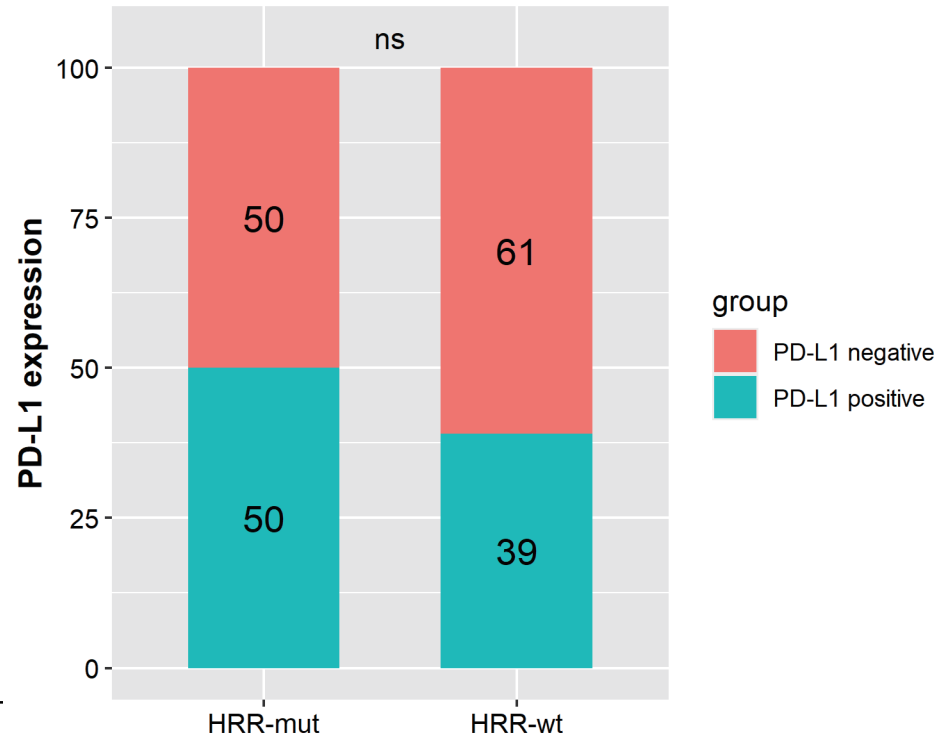**C**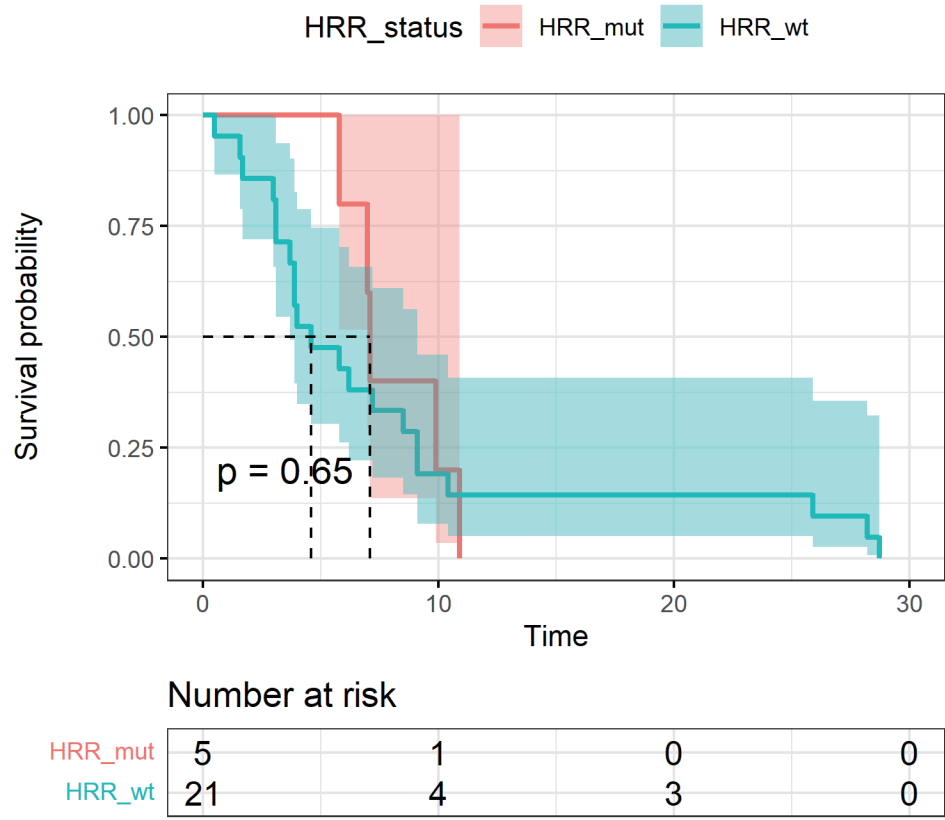**D**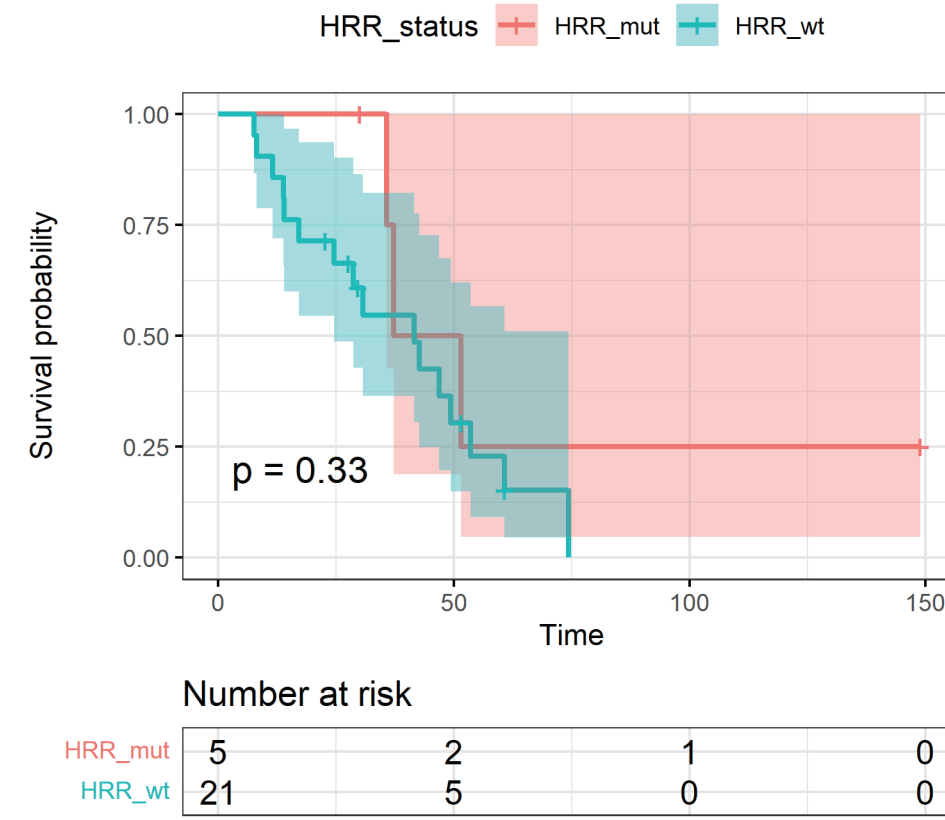

Supplement: Supplementary file 4 — Figure S4. [file CAM4-12-15304-s004.pdf]
