# Supplementary material for: Mutational pattern off homologous recombination repair (HRR)‐related genes in upper tract urothelial carcinoma
Source: Cancer Med. 2023 Jun 30;12(14):15304–16. doi: 10.1002/cam4.6175 (PMC10417099; doi:10.1002/cam4.6175)
Supplement: Supplementary file 5 — Table S1. [file CAM4-12-15304-s006.docx]

**Table S1 Demographic, clinical, and pathological data of the study population.**

| Characteristics | | ALL | HRR-mut cohort (N, %) | HRR-wt cohort (N, %) | P value |
| --- | --- | --- | --- | --- | --- |
| N |  | 197 | 74 | 123 |  |
| Age | >65 years | 109 (55.33) | 45(60.81) | 64(52.03) | 0.241 |
|  | ≤65 years | 88 (44.67) | 29(39.19) | 59(47.97) |  |
| Sex | Male | 127 (64.47) | 43(58.11) | 84(68.29) | 0.168 |
|  | Female | 70 (35.53) | 31(41.89) | 39(31.71) |  |
| Sample type | Tissue | 176 (89.34) | 66(89.19) | 110(89.43) | 1.000 |
|  | ctDNA | 22 (11.17) | 8(10.81) | 14(11.38) |  |
| Tumor location | [Pelvis](javascript:;) | 108 (54.82) | 40(54.05) | 68(55.28) | 0.879 |
|  | Ureter tract | 80 (40.61) | 31(41.89) | 49(39.84) |  |
|  | Missing | 9(4.57) | 3(4.05) | 6(4.88) |  |
